# Immunoprotection in sheep against Haemonchus contortus using its thiol-purified excretory/secretory proteins

**Published:** 2012

**Authors:** Selvarayar Arunkumar

**Affiliations:** Department of Veterinary Parasitology, Madras Veterinary College, Tamilnadu Veterinary and Animal Sciences University, Chennai, India.

**Keywords:** *Haemonchus contortus*, Excretory/secretory antigen, Antibody, Fecal egg count, Sheep

## Abstract

Excretory/Secretory antigen was prepared by culturing live adult worms of *Haemonchus contortus* in RPMI 1640 medium at a concentration of 50 worms per mL in a culture-flask at 37 ˚C for 24 hr and the culture supernatant was used as antigen. The E/S antigen was purified by thiol-sepharose affinity chromatography. On western blot analysis, it was demonstrated that thiol-purified antigen showed a single reactive band at 66 kDa. In immunization trial, sheep were administered intramuscularly with 500 µg of thiol-purified excretory/secretory antigen along with montanide as adjuvant on day 0, 30 and 60. On ELISA, it was observed that the mean absorbance values were significantly (*p* ≤ 0.01) higher up to 20 weeks post immunization in Group-I (purified antigen) compared to Group- II (unimmunized control). Further, the mean EPG values was lower in Group I (200.00 ± 40.82 to 400.00 ± 91.29) than Group II (2200.00 ± 108.01 to 5100.00 ± 169.56) and the percentage reduction in mean fecal egg counts was 88.50%. Similarly, the mean abomasal worm counts was lower in Group I (808.33 ± 78.29) than Group II (3280.00 ± 147.19) and the percentage reduction in mean abomasal worm count was 75.40%.

## Introduction

*Haemonchus contortus *is an economically important, highly pathogenic blood – feeding abomasal nematode of small ruminants, especially sheep and goats.^1^^-^^3^ It causes great economic losses in sheep industry including decreased weight gain and milk yield.^4^ The wide spread emergence of strains of* Haemonchus contortus *resistant to the currently available anthelmintics has dramatically accelerated the need to develop alternative sustainable control measures.^5^ Concern has also been raised regarding the presence of anthelmintic residues in the food chain and environment.^6^

In the recent years, much progress has been made in characterizing the protective antigens against helminthic parasites. Further, analysis of host immune response to helminthes is hampered by two main factors: (a) the complexity of antigen profiles of parasites (b) the presence of cross-reactive determinants on antigens. To identify specific antigens, excretory / secretory (E/S) products of helminthes have received increasing attention. This is due to the fact that E/S products of helminthes usually display a relatively simple antigenic composition compared to the somatic worm antigens. The E/S products play numerous roles in the host-parasite relationship such as host tissue penetration, degradation of host proteins for nourishment, etc.^7^^-^^9^ In recent years, attempts have been made to characterize the E/S products of *H. contortus *as these substances could be potential target for the immunological control of the disease. Hence, the present work was carried out to evaluate the efficacy of thiol-purified E/S antigens of *H. contortus *in inducing protection in sheep. 

## Materials and Methods

**Preparation of antigen.** Adult *Haemonchus contortus* worms were collected from abomasum of sheep slaughtered at corporation slaughter house, Perambur, Chennai. The collected worms were washed five times in normal saline and subsequently washed five times in phosphate buffered saline (PBS, Sigma, USA, pH = 7.4), containing penicillin, Sigma, USA, (500 IU mL^-1^) and streptomycin, Sigma, USA, (5 mg mL^-1^). Then, the worms were identified based on morphological features using standard keys.^10^ After thorough washing; the worms were used for preparing the antigens.

The fresh and highly motile worms were transferred to RPMI (Roswell Park Memorial Institute) 1640 medium containing penicillin (500 IU mL^-1^) and streptomycin (5 mg mL^-1^) and cultured at a concentration of approximately 50 worms per mL in a culture flask at 5 % CO_2_ atmosphere at 37 ˚C for 24 hr. The medium was changed every 6 hr after incubation and fresh medium was added with 2.00% glucose throughout incubation. Worm viability was monitored throughout this period on the basis of motility and integrity of the worms. Moreover, random samples of the culture fluid obtained during and directly after the incubation period were plated out on agar in order to exclude bacterial contaminations. After the incubation period, the culture medium was collected by decantation and filtered through a 0.22 μm filter (Millipore, Billerica, MA, USA). Then, the culture medium was centrifuged at 10000 rpm for 30 minutes at 4 ˚C and the supernatant was labeled as E/S antigen. Finally, the antigen obtained was concentrated by dialysis (membrane cut off, 12 kDa) against polyethylene glycol (PEG 6000 – SRL, Mumbai, - India) over a period of 6 hr and stored at -20 ˚C with 0.02 % sodium azide as preservative till further use. The protein concentration of the E/S antigen was determined using bicinchoninic acid (BCA) method using protein estimation kit.^11^

**Purification of E/S antigen. **Affinity chromatography was carried out for purification of E/S antigen.^12^ The thiol- sepharose packed column (5 cm bed height) was equilibrated with equilibration buffer (pH = 7.4) at a flow rate of 5 mL per hour. Protein sample (10 mg) was applied onto a column. The unbound fractions were eluted by washing the column with 10 bed volumes of equilibration buffer till the optical density (OD) value at 280 nm returned to a steady base line. Then, the thiol-bound fractions were eluted in elution buffer (10 mM Tris, 0.5 M NaCl, 50 mM DTT) in 1 mL fractions at a flow rate of 5 mL per hour. Based on the OD value at 280 nm, the peak fractions were pooled and concentrated by dialysis against PEG 6000. The purified antigen was stored at -20 ˚C till further use. The Protein concentration of the purified E/S antigen was determined (BCA kit, Genei, Bangalore). 

**Characterization of E/S antigen. **Sodium dodecyl sulphate polyacrylamide gel electrophoresis (SDS-PAGE) was carried out to observe the polypeptide patterns of thiol-purified E/S antigen of *H. contortus* under reducing gel conditions.^13^ The immunogenic fraction was identified by western blot analysis using mini trans-blot electro-phoretic transfer cell.^14^ (BioRad, Hercules, CA, USA). After the electrophoretic run, the nitrocellulose membrane was removed and was blocked in blocking buffer at 37 ˚C for 2 hr. Then, the membrane was washed in washing buffer three times with gentle agitation for 5 minutes each. The membrane was incubated in sheep immune serum at a dilution of 1:100 in PBS for 2 hr at 37 ˚C with gentle shaking. The nitrocellulose membrane was washed in washing buffer three times for 5 minutes each and immersed in solution of 1:1000 diluted anti- sheep IgG HRP conjugate (Sigma Chemical Company, St. Louis, MO, USA) in PBS for 2 hr at 37 ˚C. Then the membrane was washed three times in washing buffer for 5 minutes each. The membrane was treated with substrate solution diaminobenzidine (DAB) till the bands appeared. 

**Immunization Trial. **Immunization trial was conducted in sheep to evaluate the efficacy of thiol-purified E/S antigen of *H. contortus*. Twelve Madras red breeds of male sheep aged around eight months old were procured from Livestock Research Station, Kattupakkam, Tamil Nadu. All the sheep were maintained in clean animal shed and were fed with concentrate mixture (200 g per animal), green fodder and water *ad* *libitum *during the research period. All animals were dewormed with ivermectin at the dose rate of 0.2 mg kg^-1^ 21 days prior to start of experiment. Sheep were divided into two groups; each group consisted of six sheep. In group I, sheep were immunized with 500 µg of affinity purified E/S antigen along with adjuvant montanide on days 0, 30 and 60 through intramuscular route. In group II, sheep were used as the unimmunized control. Experimental animals were maintained as per the guidelines issued by the institutional animal ethical committee. 

**Standardization of ELISA. **Serum antibody responses in sheep immunized with affinity purified E/S antigen was evaluated by enzyme linked immunosorbent assay (ELISA). Mono-specific positive serum was obtained from Division of Parasitology, Indian Veterinary Research Institute, Izat nagar. Negative serum was collected from a healthy 2 to 3 weeks old Madras red breed of lamb from Livestock Research Station, Kattupakkam (TANUVAS) Tamilnadu. Test serum samples were collected at weekly intervals from all the sheep from 0 to 21 weeks of experimental period. 

The optimum concentration of antigen, serum and conjugate (anti-sheep IgG/HRP, Sigma Chemical Company, St. Louis, MO, USA) were determined by checker board titration method using serial dilution of antigen, serum and conjugate. Four different concentrations of antigen (namely 1 µg mL^-1^, 2 µg mL^-1^, 5 µg mL^-1^, 7 µg mL^-1^, diluted in carbonate-bicarbonate buffer) were added to the 96 well flat bottom ELISA plate. Serial dilutions of serum (known positive and negative) namely 1 in 100, 1 in 200, 1 in 400, 1 in 800, 1 in 1600 and 1 in 3200 were used. The conjugate was checked in dilutions of 1 in 1000, 1 in 2000, 1 in 5000 and 1 in 10000. The test was conducted and the OD (optical density) values were obtained for each concentration. The maximum difference in OD value obtained between positive and negative serum was selected as the optimum dilution for use. The optimum concentration of antigen (2 µg mL^-1^), serum (1 in 200) and the conjugate (1 in 5000) was arrived by this method which was used in the test procedure. ELISA was carried out following the standard method. ^15^

**Assessment of worm burden: Copro-culture. **The dung sample was cultured for the recovery of third stage infective larvae (L_3_) of *H. contortus *with certain modifications.^16^ The dung was collected from the experimental sheep and was subjected to 60 ˚C for 1 hour in hot air oven to kill the eggs if any. The live adult female *H. contortus *worms were teased and triturated well in pestle and mortar and it was mixed with sterile dung.

Wide mouthed glass jar was filled with fecal mixture, closed with the lid and incubated for 7 days at room temperature. Fungi are sometimes troublesome but indicate that the conditions in the culture are ideal. The growth of the fungi was reduced by stirring the culture each day. After 7 days of incubation, the lukewarm water was poured into the side of the culture jar and it was kept inverted position for 1 hour. Then, the larvae were recovered from the brim of the culture jar using Pasteur pipette. Larvae were identified based on the shape, total length, breadth, length of the esophagus, number and shape of the intestinal cells, shape of the larval tail, relative size of the tail sheath.^17^^,^^18^

**Fecal egg count.** A total of 5000 infective larvae (L_3_) of *H. contortus* were given orally to individual sheep on 90^th^ day after immunization. The dung samples were collected from the rectum of each sheep from 21 days after challenge at weekly intervals up to 8 weeks for egg count. The fecal egg count was estimated by modified McMaster method.^19^ The number of eggs counted was multiplied by 100 to arrive at the number of eggs per gram (EPG) of feces. The level of egg reduction, expressed as percentage, was calculated by using the following formula: 

$\frac{\mathrm{Mean EPG of control}–\mathrm{Mean EPG of immunized group}}{\mathrm{Mean EPG of control}}\times 100$

**Abomasal worm counts. **All the experimental sheep were slaughtered on day 60 after challenge infection.^20^ The abomasum was tied off and removed intact. Then, it was opened along the lesser curvature and flooded with normal saline. The contents were washed again with normal saline and the supernatant fluid was discarded until clear sediment was obtained. Then, the adult worms were collected from the sediment. The adult worms present in the abomasum were counted manually. The percentage of total worm reduction was calculated using the following formula:

Mean worm count of control – Mean worm count of immunized group Mean worm count of control×100

**Statistical analysis. **The data obtained in the present study were statistically analyzed by SPSS software (version 13, SPSS Inc., Chicago, IL, USA).

## Results

In the present investigation, a total of 10,000 live adult *Haemonchus* *contortus* worms were collected from the abomasum of sheep. During *in vitro* culture, adult worms remained viable as assessed qualitatively by both motility and clumping tendency. No bacterial contamination was also detected during incubation. The protein concentration of E/S antigen was 1.50 mg mL^-1^.

In the present study, the E/S antigen of *H. contortus *was further purified by thiol-sepharose affinity chromatography. The thiol-bound fractions were eluted using elution buffer and the peak fractions (47 to 53) were pooled and concentrated by dialysis against PEG 6000. The elution profile of E / S antigen showed two minor peaks in unbound fractions and one major peak in bound fractions. The protein content of thiol-purified E/S antigen was found to be 1.60 mg mL^-1^. On characterization of purified E/S antigen by SDS- PAGE revealed a single band at 66 kDa. On western blot analysis, the affinity purified E/S antigen probed with serum from sheep infected with *H. contortus* showed a strong reactive band at 66 kDa. 

Further, the assessment of serum antibody levels were monitored in immunized and control sheep at weekly intervals by ELISA. In Group-I, the mean absorbance values gradually increased from second weeks post immunization and reached a peak value of 1.26 ± 0.05 on eighth week post immunization. The serum antibody levels in affinity purified group were significantly (*p* ≤ 0.01) higher and was maintained up to 20 weeks post immune-ization compared to unimmunized control group whereas the mean absorbance values was low (0.09 ± 0.01 to 0.16 ± 0.01) throughout the observation period in Group II (unimmunized control). This study revealed that the mean absorbance values of Group-I were significantly (*p* < 0.01) higher than Group-II throughout the observation period. 

The results of the fecal egg counts are presented in Table 1. In Group-I, the mean EPG values were significantly low (200.00 + 40.82 to 400.00 + 91.29) up to 42^nd^ day after challenge and thereafter gradual increase noticed up to 70^th^ day after challenge infection (500.00 + 91.29 to 700.00 + 93.49) whereas in control group, the mean EPG values had increased gradually and were maintained high up to day 70 (2200.00 + 108.01 to 5100.00 + 169.56). The mean EPG values were lower in Group-I compared to Group- II**. **The percentage reduction in mean fecal egg counts was found to be 88.50%. This study clearly revealed that there was a highly significant (*p* < 0.01) reduction in mean EPG values in immunized groups compared to the control group.

**Table 1:** EPG values of immunized and control sheep (Mean ± SE).

**Days Post Challenge**	**Group I** * **(Purified)**	**Group II ** **(Unimmunized Control)**
**21**	300.00 ± 40.82	2200.00 ± 108.01
**28**	200.00 ± 40.82	3200.00 ± 248.33
**35**	400.00 ± 91.29	3400.00 ± 226.38
**42**	300.00 ± 70.71	3700.00 ± 45.64
**49**	500.00 ± 91.29	4400.00 ± 176.78
**56**	600.00 ± 70.71	5100.00 ± 169.56
**63**	650.00 ± 91.29	4900.00 ± 188.19
**70**	700.00 ± 93.49	4800.00 ± 142.89
**Analysis of variance**		
**Source**	**Degree of Freedom**	**F-value**
**Groups**	1	125.55*
**Periods**	7	2.98*

* indicates highly significant (*p* < 0.01).

It was also observed that the worms recovered from immunized animals appeared pale, thin and stunted in growth when compared to those recovered from the control animals. In Group-I, the mean abomasal worm counts were significantly lower (808.33 + 78.29) compared to the control group. Whereas, the mean abomasal worm counts was higher in control group (3280.00 + 147.19). The percentage reduction in mean abomasal worm counts was found to be 75.40%.

## Discussion

Excretory / Secretory products of helminthes have received increasing attention recently. This is due to the fact that E/S products of helminthes usually display a relatively simple antigenic composition compared to the somatic worm antigens. Further, they play numerous roles in the host-parasite interactions. In the present study, *in vitro *culture of live adult worms was made and the E/S antigen was isolated. Similar culture procedures were reported by several workers for isolating the E/S antigen from *Haemonchus* *contortus.*^21^^-^^24^ The protein concentration obtained in this study is in accordance with the reports of previous workers.^25^^,^^26^

Thiol-binding protein fractions of E/S and membrane proteins of parasitic nematodes have been shown to contain protective properties. Theoretically, all proteins containing reduced cysteine residues can bind covalently to thiol-sepharose. The reactivity and accessibility of the SH-groups is determined by their sequence context and location within the tertiary structure, which also determines the ease by which bound proteins can be eluted. In addition, proteins may stick non-covalently with high affinity to bound proteins although the binding and the washing were done in a high-ionic strength (0.50 M NaCl) to reduce such binding. Therefore, thiol-binding fractions of *H. contortus *have been shown to be enriched in cysteine protease activity. In the present study, the protein content of thiol-purified antigen was 1.60 mg mL^-1^ and the elution profile showed one major peak. This finding is compatible with the reports of earlier workers who observed one major peak and two minor peaks in elution profile.^27^

On western blot analysis, a single band at 66 kDa was detected in thiol-purified antigen. These findings are in accordance with the reports of previous workers.^28^^,^^29^ In this trial, sheep were immunized with 500 µg of affinity purified E/S antigen (Aff-66 kDa) along with montanide on day 0, 30 and 60 intramuscularly. The adjuvant used in the present study produced persistent antibody titers, without causing any tissue reactions at injection site. Several investigators conducted immunization trials with different dose schedule in sheep using E/S antigen. Serum antibody levels in Group I might be due to the effect of administration of thiol-purified antigen which induced strong antibody responses. Similar observations were made by many researchers. A significant increase in serum antibody titers were observed in affinity purified protein immunized group.^30^

In the present study the mean EPG values significantly lower in Group I compared to Group II and the percentage reduction in mean fecal egg count was 88.50%. Many workers reported different percentage of mean fecal egg count in immunized animals. Sheep vaccinated with purified E/S antigen induced significant reduction (> 70%) in mean fecal egg counts compared to the non-vaccinated control group. Sheep vaccinated with a thiol-binding fraction of E/S protein showed a significant reduction in fecal egg output of 52.00 % and abomasal worm burden of 50.00%. A reduction in fecal egg output of more than 50.00% was observed in vaccinated sheep. 

This study further revealed that the mean abomasal worm count was significantly lower in Group I compared to Group II. Whereas, the mean abomasal worm counts was higher in control groups. The percentage reduction in mean abomasal worm count was found to be 75.40%.

Several workers reported different percentage of mean abomasal worm counts in immunized animals. A significant reduction in abomasal worm burden of 50.00% was reported in vaccinated sheep compared to control group. A reduction in abomasal worm count of 46.00% was observed in vaccinated sheep. 

In the present study, no immature worms could be recovered from the abomasal mucosa of immunized animals. This finding is in agreement with the reports of previous workers who reported no arrested larvae and immature worms in the abomasal mucosa of immunized sheep.^31^ The variations observed in reduction percentage of mean fecal egg count and abomasal worm count in the present study might be due to the nature of antigen, method of purification followed, dosage of antigen, type of adjuvant used. Based on the results of the present study, it was observed that immunization with thiol-purified E/S antigen produced immune effect on female worm and it was operating before sexual maturity. Because of the immunizing effect, female worms were stunted in their development which could reduce their egg laying capacity. Hence in the present study, protection levels were higher in terms of reduction in fecal egg counts than in abomasal worm counts.

Based on the results, it was concluded that the thiol-purified E/S antigen induced significantly higher protection levels in terms of reduction in mean fecal egg counts (88.50%) and reduction in mean abomasal worm counts (75.40%). Further, it is indicated that the thiol-purified E/S antigen are highly immunogenic in nature and could be useful for protection against haemonchosis in sheep.
